# Mechanisms underlying the role of the oral-gut-liver axis in the promotion of liver fibrosis by periodontal disease: an interacting network of inflammation, microbiota, and barrier function

**DOI:** 10.3389/fcimb.2026.1942882

**Published:** 2026-09-18

**Authors:** Shuxin Li, Zihan Gao, Yutao Ma, Jingqi Zhang, Wei Wei

**Affiliations:** 1 Department of Urology, The First Hospital of Jilin University, Changchun, China; 2 Department of Urology, Shengjing Hospital, China Medical University, Shenyang, China

**Keywords:** hepatic stellate cell, inflammation, intestinal barrier, liver fibrosis, microbiota, oral-gut-liver axis, periodontal disease, toll-like receptor

## Abstract

Periodontal disease is an independent risk indicator for the progression of liver fibrosis, yet the underlying mechanisms remain incompletely defined. Emerging evidence suggests that the “oral-gut-liver axis” plays a critical role. This review aims to systematically synthesize the molecular mechanisms that may plausibly contribute to the pathogenesis of liver fibrosis via this axis and to propose a novel spatiotemporal framework to guide future research and therapeutic strategies. We propose a three-phase framework that temporally dissects the fibrogenic process into initiation, amplification, and maintenance phases—a distinction that, to our knowledge, has not been systematically applied to the oral-gut-liver axis in previous literature. This framework is proposed as a working hypothesis based on integrating current preclinical and observational evidence and is intended to guide future mechanistic and interventional studies. In the initiation phase, periodontal pathogens and their virulence factors disseminate haematogenously or via the intestinal route to the liver, where, in animal models, they have been shown to activate Toll-like receptor (TLR)/nuclear factor-κB (NF-κB) signaling and trigger the initial inflammatory response. In the amplification phase, hepatic inflammatory cytokines disrupt the intestinal barrier, leading to a “leaky gut” and massive endotoxin translocation into the liver, theoretically establishing a self-perpetuating positive feedback loop that may drive Kupffer cell M1 polarization and hepatic stellate cell (HSC) activation. In the maintenance phase, even after the oral trigger subsides, liver-intrinsic mechanisms—including HSC autocrine TGF-β signaling, epigenetic reprogramming, extracellular matrix crosslinking, liver sinusoidal endothelial cell capillarization, and bile acid–farnesoid X receptor (FXR)/Takeda G protein-coupled receptor 5 (TGR5) dysregulation—are hypothesized to sustain and contribute to the aggravation of fibrosis, based largely on preclinical evidence. Despite promising preclinical and observational evidence, translation to clinical practice remains hampered by the lack of high-quality randomized controlled trials with histological reversal as a hard endpoint. Future research should integrate multi-omics analysis, organoid and humanized animal models, and rigorously designed interventional trials to validate the oral-gut-liver axis as a therapeutic target. This review provides an integrated mechanistic framework that may inform future opportunities for early prevention and multidisciplinary management of periodontal disease-associated liver fibrosis.

## 1 Introduction

Periodontal disease is a chronic, infectious, and inflammatory condition driven by specific pathogens. It primarily affects the tooth-supporting tissues, including the gums, periodontal ligament, alveolar bone, and cementum. Clinical features include gum redness and swelling, bleeding on probing, periodontal pocket formation, clinical attachment loss, and progressive alveolar bone resorption. In advanced stages, the disease can lead to tooth mobility and even tooth loss. Based on lesion severity, periodontal disease is classified into two main stages: gingivitis, which is confined to the gingival tissues, and periodontitis, which involves the deeper periodontal tissues (Nocini et al., 2020; Luchian et al., 2021; Yadalam et al., 2022; Palideh et al., 2023; Higashi and Taguchi, 2025). The core pathogenesis of this disease lies in an imbalance in the host’s immune-inflammatory response to pathogenic microorganisms within the subgingival plaque biofilm, disrupting the dynamic balance between tissue destruction and repair (Luchian et al., 2021; Kozak and Pawlik, 2023; Palideh et al., 2023). Epidemiological studies show that periodontal disease is one of the most prevalent oral diseases worldwide, affecting approximately 20%–50% of adults, with some estimates exceeding 1 billion affected individuals (Amato et al., 2022; Soheili et al., 2024; Hashim et al., 2025; Wu et al., 2026). In the United States, nearly half of adults aged 30 and older have periodontal disease to varying degrees, with significant racial disparities in prevalence (Fleming et al., 2025; Moreno et al., 2026). Given its high prevalence and continuing upward trend, periodontal disease has become a public health challenge that cannot be ignored (Kopić et al., 2019; Agarwal et al., 2025). This oral–systemic interaction has driven the emergence of periodontal medicine, further emphasizing the critical role of oral health in maintaining overall health (Yoshida et al., 2021; Ungureanu et al., 2023).

Liver fibrosis is a pathological reparative response to chronic liver injury. It is characterized by an imbalance between extracellular matrix (ECM) synthesis and degradation, resulting in excessive accumulation of fibrous tissue. This gradually replaces normal parenchyma, distorts hepatic architecture, and impairs liver function (Delgado et al., 2021; Lucantoni et al., 2021; Li and Zhu, 2022). This pathological process can be driven by various causes, including viral hepatitis, alcohol abuse, metabolic dysfunction-associated steatotic liver disease (MASLD), autoimmune liver disease, and drug-induced liver injury (Yao et al., 2022; Zhang et al., 2022; Yang et al., 2026). At the molecular level, persistent hepatocyte injury activates hepatic stellate cells, accompanied by the release of inflammatory cytokines, infiltration by immune cells and fibroblasts, and dysregulation of key signaling pathways, such as TGF-β, collectively driving extracellular matrix metabolic imbalance (Yin et al., 2019; Li et al., 2023; Cerrito et al., 2025). Early-stage liver fibrosis is, to some extent, reversible; however, if the underlying cause persists or intervention is delayed, the condition progresses to an irreversible stage (Zhang et al., 2016; Yin et al., 2019; Li et al., 2023). Epidemiologically, as the central pathological hub in the progression of various chronic liver diseases (CLD), liver fibrosis imposes a substantial global burden. Chronic liver disease causes approximately 2 million deaths worldwide each year, accounting for about 4% of total mortality, with fibrosis observed across a wide spectrum of etiologies including viral hepatitis, alcohol-related liver disease, and MASLD (Yamaguchi, 2026; Yang et al., 2026). Although precise prevalence data in the general population are currently lacking, multiple studies have confirmed that this condition is prevalent among patients with chronic liver disease and represents a critical pathological stage that determines prognosis, posing a substantial challenge to global public health (Zhang et al., 2022; Tsuchiya et al., 2024). If left uncontrolled, liver fibrosis leads to serious clinical consequences. Widespread deposition of fibrous tissue disrupts liver lobule structure, forming regenerative nodules and causing hemodynamic disturbances. These changes ultimately progress to cirrhosis (Iredale et al., 2017; Matsuda and Seki, 2020). The persistent fibrotic microenvironment significantly increases the risk of hepatocellular carcinoma (HCC), serving as a critical pathological bridge between cirrhosis and malignant transformation (Nan et al., 2022; Tang et al., 2025). As functional units of the liver parenchyma are lost, the liver’s synthetic, detoxification, and metabolic functions decline comprehensively, potentially leading to life-threatening complications associated with portal hypertension, such as ascites, hepatic encephalopathy, and bleeding from ruptured esophageal or gastric varices (Lee et al., 2016; Iredale et al., 2017). Furthermore, the severity of fibrosis is significantly positively correlated with the risk of all-cause mortality, cardiovascular events, and liver-related adverse events such as liver decompensation (Roskilly et al., 2021; Zhou et al., 2026a; Zhou et al., 2026b).

In recent years, a growing body of evidence has shown that periodontal disease and liver fibrosis are not isolated conditions but share significant epidemiological overlaps and clinical associations. The prevalence of liver fibrosis is significantly higher among patients with periodontal disease, while patients with chronic liver disease generally have poorer oral health (Vegda et al., 2024; Joo et al., 2025). This comorbidity suggests a potentially common pathophysiological link between the two conditions. However, the mechanistic basis of this association—particularly how a localized oral inflammatory condition could remotely drive hepatic fibrogenesis—remains incompletely understood. Preclinical and observational studies show that periodontal pathogens, their toxins, and inflammatory mediators can reach the liver directly via the bloodstream; however, a more critical pathway involves the “oral-gut-liver axis” (Kuraji et al., 2021). Dysbiosis and local inflammation from periodontal disease can alter the gut microbiota, compromise intestinal barrier function, and increase translocation of enteric endotoxins, which then enter the liver via the portal vein, activate hepatic immune cells and hepatic stellate cells, and accelerate fibrosis (Sun et al., 2025; Xiang and Hua, 2026). Concurrently, periodontal disease induces systemic low-grade inflammation. This inflammatory state further exacerbates hepatic inflammation and fibrosis through pro-inflammatory factors such as tumor necrosis factor-α (TNF-α) and interleukin-6 (IL-6) (Sun et al., 2025; Xiang and Hua, 2026). Against this backdrop, this review aims to systematically synthesize the molecular mechanisms by which periodontal disease promotes liver fibrosis through the oral-gut-liver axis. Rather than enumerating isolated pathways, we propose an integrated three-phase framework—encompassing initiation, amplification, and maintenance—to organize the mechanistic evidence. The initiation phase covers how periodontal pathogens disseminate systemically and activate hepatic TLR/NF-κB signaling, producing initial pro-inflammatory cytokines. The amplification phase explains how these inflammatory signals disrupt intestinal barrier function, creating a “leaky gut” that increases endotoxin delivery to the liver and may promote Kupffer cell M1 polarization and hepatic stellate cell (HSC) activation, creating a self-perpetuating loop, based on evidence from animal models. The maintenance phase describes how, even after the initial oral trigger diminishes, liver-intrinsic mechanisms—including HSC autocrine TGF-β signaling, epigenetic reprogramming of pro-fibrotic genes, extracellular matrix crosslinking, liver sinusoidal endothelial cell capillarization, and bile acid–FXR/TGR5 dysregulation—may sustain the activated HSC phenotype and contribute to progressive fibrosis. This framework integrates current evidence and identifies phase-specific intervention points for future clinical translation.

It should also be noted that the mechanistic evidence reviewed in the following sections originates from three distinct levels of scientific evidence—ranging from directly demonstrated mechanisms derived from animal models or well-controlled *in vitro* experiments, through plausible pathways inferred from observational human studies and indirect biomarkers, to speculative models that remain to be tested experimentally. We have deliberately distinguished these levels throughout the manuscript by using precise language that reflects the strength and nature of the underlying evidence, and we have explicitly indicated where direct human evidence is lacking. This transparent stratification is intended to help readers assess confidence in each proposed mechanism and identify priority areas for future validation studies. The proposed framework is derived predominantly from preclinical mechanistic studies and clinical observational data. While these lines of evidence are convergent and compelling, they do not yet establish a direct causal chain in humans. Therefore, the mechanistic pathways and epidemiological associations discussed in the following sections should be interpreted as a plausible biological model that requires rigorous validation in future interventional studies.

While the concept of an “oral-gut-liver axis” has been recognized in previous reviews, most existing frameworks describe this axis as a non-temporal, linear sequence in which oral dysbiosis leads to alterations in the gut microbiota and endotoxin translocation, which in turn drive hepatic inflammation and fibrosis (Lyu et al., 2025). These models do not temporally distinguish the phases of fibrogenesis, nor do they differentiate early, potentially reversible, inflammation-driven activation of hepatic stellate cells (HSCs) from the later, self-sustaining fibrotic state maintained by liver-intrinsic mechanisms (Wu et al., 2026). By contrast, our three-phase framework—encompassing initiation, amplification, and maintenance—provides a dynamic, mechanistic dissection of the fibrogenic cascade. This temporal stratification enables us to identify the critical transition point at which a reversible inflammatory response becomes an irreversible fibrotic program, elucidate how a self-perpetuating positive feedback loop between hepatic inflammation and intestinal barrier dysfunction is established and sustained, and propose phase-specific therapeutic intervention strategies not addressed by existing non-temporal models. To our knowledge, no previous review has integrated the available mechanistic evidence into such a dynamic, temporally stratified framework for periodontal disease-associated liver fibrosis.

The proposed three-phase framework is a mechanistic synthesis and working hypothesis derived from integrating currently available preclinical and observational evidence, rather than a definitively established pathogenic cascade in humans. While this framework provides a useful heuristic for organizing the complex interactions along the oral-gut-liver axis and for identifying phase-specific intervention points, it remains to be rigorously tested in future prospective studies and interventional trials. Readers are therefore encouraged to interpret the proposed model as a conceptual guide for future research, not as a proven sequence of events.

### 1.1 Search strategy and evidence synthesis

This narrative review was informed by a systematic literature search of PubMed, Web of Science, and Scopus databases up to March 2026, without language restrictions. The search strategy combined keywords related to periodontal disease (“periodontitis,” “periodontal disease,” “Porphyromonas gingivalis”), liver fibrosis (“liver fibrosis,” “hepatic fibrosis,” “cirrhosis,” “hepatic stellate cell”), the gut–liver axis (“gut–liver axis,” “intestinal barrier,” “endotoxin,” “bile acid”), and mechanistic pathways (“TLR,” “NF-κB,” “TGF-β,” “TGR5,” “FXR”). We manually screened the reference lists of included articles and relevant reviews to identify additional studies. Given the broad, multidisciplinary nature of this topic, we used a narrative synthesis approach rather than a formal meta-analysis. We included studies that addressed mechanistic, preclinical, or clinical aspects of the association between periodontal disease and liver fibrosis via the oral–gut–liver axis. We deliberately stratified the evidence into three levels (directly demonstrated in animal/human studies, inferred from observational data, and speculative) as presented in Section 6.1, to provide readers with a transparent assessment of the strength of the underlying data. This stratification was applied during synthesis, not as a formal exclusion criterion during screening.”

## 2 Clinical characteristics and phenotypic associations between periodontal disease and liver fibrosis: clinical foundation for the three-phase framework

Epidemiological and clinical studies have gradually revealed a phenotypic association between periodontal disease and liver fibrosis, which is particularly pronounced in the context of nonalcoholic fatty liver disease (NAFLD). Multiple cross-sectional and cohort studies have shown a dose-response relationship between periodontal disease severity and liver fibrosis stage; this association remains partially independent even after adjustment for metabolic confounders, suggesting a biological link beyond shared risk factors.

### 2.1 A dose-response relationship: epidemiological and interventional evidence linking periodontitis severity to liver fibrosis stage

Periodontal disease and liver fibrosis are highly prevalent chronic conditions that share common risk factors and pathophysiological mechanisms. Recent epidemiological and clinical studies have shown a significant positive association between the two. Both conditions have high prevalence rates and share common pathophysiological mechanisms, including insulin resistance, metabolic syndrome, and systemic inflammation (Lyu et al., 2025; Mousa et al., 2026). Several large-scale population-based cohort studies have consistently shown that the severity of periodontitis is associated with a dose-response relationship regarding the risk of liver disease. For example, the Finnish Health 2000 study, which conducted long-term follow-up of 6,165 individuals without liver disease at baseline, found that the risk ratio for severe liver disease events among patients with severe periodontitis was 3.69; this association remained significant even after fully adjusting for alcohol intake, smoking, metabolic risk factors, and socioeconomic factors (Helenius-Hietala et al., 2019). Data from the UK Biobank, based on 13 years of follow-up among approximately 500,000 participants, further confirmed that poor oral health is significantly associated with an increased risk of metabolic dysfunction-associated steatotic liver disease (MASLD) (HR = 1.42), with 19.4% of this effect mediated by metabolic pathways involving very-low-density lipoproteins, fatty acids, and triglycerides (Ye et al., 2026).

Among individuals with diagnosed liver disease, the prevalence and severity of periodontal disease are closely associated with the stage of liver fibrosis. Clinical observations show that the prevalence of periodontitis is significantly elevated in patients with cirrhosis, and the severity of periodontitis is independently associated with cirrhosis (Rinčić et al., 2025). Studies of patients with nonalcoholic fatty liver disease (NAFLD) have also found a significant association between periodontitis and the stage of liver fibrosis; this association remained robust after adjusting for metabolic syndrome factors, indicating an effect beyond traditional metabolic risk factors (Alazawi et al., 2017). A 5-year follow-up study involving 341 NAFLD patients further quantified this relationship: patients with higher levels of clinical attachment loss (CAL) had an increased risk of liver fibrosis; this association was even more pronounced in the obese subgroup (Kuroe et al., 2021). Furthermore, periodontal probing depth ≥ 6 mm was significantly associated with an NAFLD fibrosis score (NFS) > 0.675, suggesting that the extent of periodontal tissue destruction may serve as a clinical indicator of liver fibrosis risk (Hiroshimaya et al., 2026). Autopsy studies have also found a positive correlation between the extent of tooth loss and the severity of liver disease, providing pathological evidence for this association (Kuraji et al., 2024). Interventional studies and natural cohort data provide indirect evidence of a causal relationship. An interventional study on periodontal treatment in patients with cirrhosis found that periodontal treatment improved Model for End-Stage Liver Disease (MELD) scores, systemic inflammatory markers, and cognitive function, while reducing endotoxin and lipopolysaccharide-binding protein levels (Bajaj et al., 2018). The U.S. Veterans Compensation Cirrhosis Cohort Study showed that patients who received regular preventive dental and periodontal maintenance services had significantly reduced risks of liver decompensation, hepatocellular carcinoma, and hospitalization, and that this protective effect was independent of other medical interventions (Bajaj et al., 2026).

**Table 1** summarizes key epidemiological and interventional studies, including their populations, sample sizes, main findings, and limitations. In summary, a wealth of epidemiological, clinical staging, and interventional evidence collectively supports the view that periodontal disease is associated with the progression of liver fibrosis and may serve as a modifiable source of systemic inflammation. Its severity is clearly positively correlated with the stage of liver fibrosis, laying a solid clinical foundation for further exploration of the underlying mechanisms (Ezhilarasan, 2021; Dioguardi et al., 2025). However, these observations still cannot prove that changes in periodontal conditions will reverse or halt liver fibrosis in affected patients, highlighting the need for interventional trials targeting histological endpoints.

**Table 1:** Summary of key epidemiological and interventional studies linking periodontal disease to liver fibrosis.

Study	Population	Sample size	Liver disease	Key findings	Key limitations
(Helenius-Hietala et al., 2019)	Finnish general population (liver-disease-free at baseline)	6,165	Incident severe liver disease	Severe periodontitis associated with 3.69-fold higher risk of severe liver events; independent of alcohol, smoking, and metabolic factors.	Observational; fibrosis not directly assessed; limited events.
(Ye et al., 2026) (UK Biobank)	UK general population	~500,000	MASLD	Poor oral health linked to HR 1.42 for MASLD; 19.4% mediated by VLDL, fatty acids, and triglycerides.	Self-reported oral health; no standardized periodontal exam; residual confounding.
(Alazawi et al., 2017)	NAFLD patients from hepatology clinic	84	NAFLD	Periodontitis is associated with significant fibrosis (≥F2) independent of metabolic syndrome.	Cross-sectional; small sample; single center.
(Kuroe et al., 2021)	Japanese adults with NAFLD	341	NAFLD	Higher CAL is associated with increased fibrosis risk over 5 years; the association is stronger in the obese subgroup.	Observational; fibrosis assessed by non-invasive scores (NFS/FIB-4); single-center.
(Hiroshimaya et al., 2026)	Japanese patients with suspected NAFLD	NR	NAFLD	Probing depth ≥6 mm associated with high NFS (>0.675), suggesting periodontal destruction as a fibrosis indicator.	Cross-sectional; non-invasive fibrosis assessment only; selection bias possible.
(Rinčić et al., 2025)	Patients with cirrhosis vs. controls	123 (81 cirrhosis, 42 controls)	Cirrhosis	Periodontitis severity independently associated with cirrhosis; correlated with Child-Pugh/MELD.	Cross-sectional; small sample; controls not well-matched.
(Bajaj et al., 2018)	Cirrhosis patients receiving SRP	40	Cirrhosis	SRP improved MELD scores, reduced serum LPS/LBP, and improved cognition over 3 months.	Uncontrolled; small; short follow-up; surrogate endpoints.
(Bajaj et al., 2026) (Veterans Cohort)	Veterans with cirrhosis	Large cohort (claims data)	Cirrhosis	Regular preventive dental care is linked to dose-dependent reductions in decompensation, HCC, and hospitalization; independent of other interventions.	Observational claims data; confounding by health-seeking behavior; no histological assessment.

BOP, bleeding on probing; CAL, clinical attachment loss; CPI, Community Periodontal Index; HCC, hepatocellular carcinoma; HR, hazard ratio; LBP, lipopolysaccharide-binding protein; LPS, lipopolysaccharide; MASLD, metabolic dysfunction-associated steatotic liver disease; MELD, Model for End-stage Liver Disease; NAFLD, nonalcoholic fatty liver disease; NFS, NAFLD fibrosis score; PPD, periodontal probing depth; RR, risk ratio; SES, socioeconomic status; SRP, scaling and root planing; VLDL, very-low-density lipoprotein; MELD, Model for End-stage Liver Disease.

## 3 Initiation phase: periodontal pathogen dissemination and hepatic TLR/NF-κB activation

This section addresses the initiation phase of the fibrogenic cascade by examining how periodontal pathogens—particularly the red complex—disseminate from oral niches into the systemic circulation and how their components activate hepatic Toll-like receptor signaling. First, this phase establishes the microbial trigger that subsequently initiates hepatic inflammation and intestinal barrier disruption (**Figure 1**).

**Figure 1:**
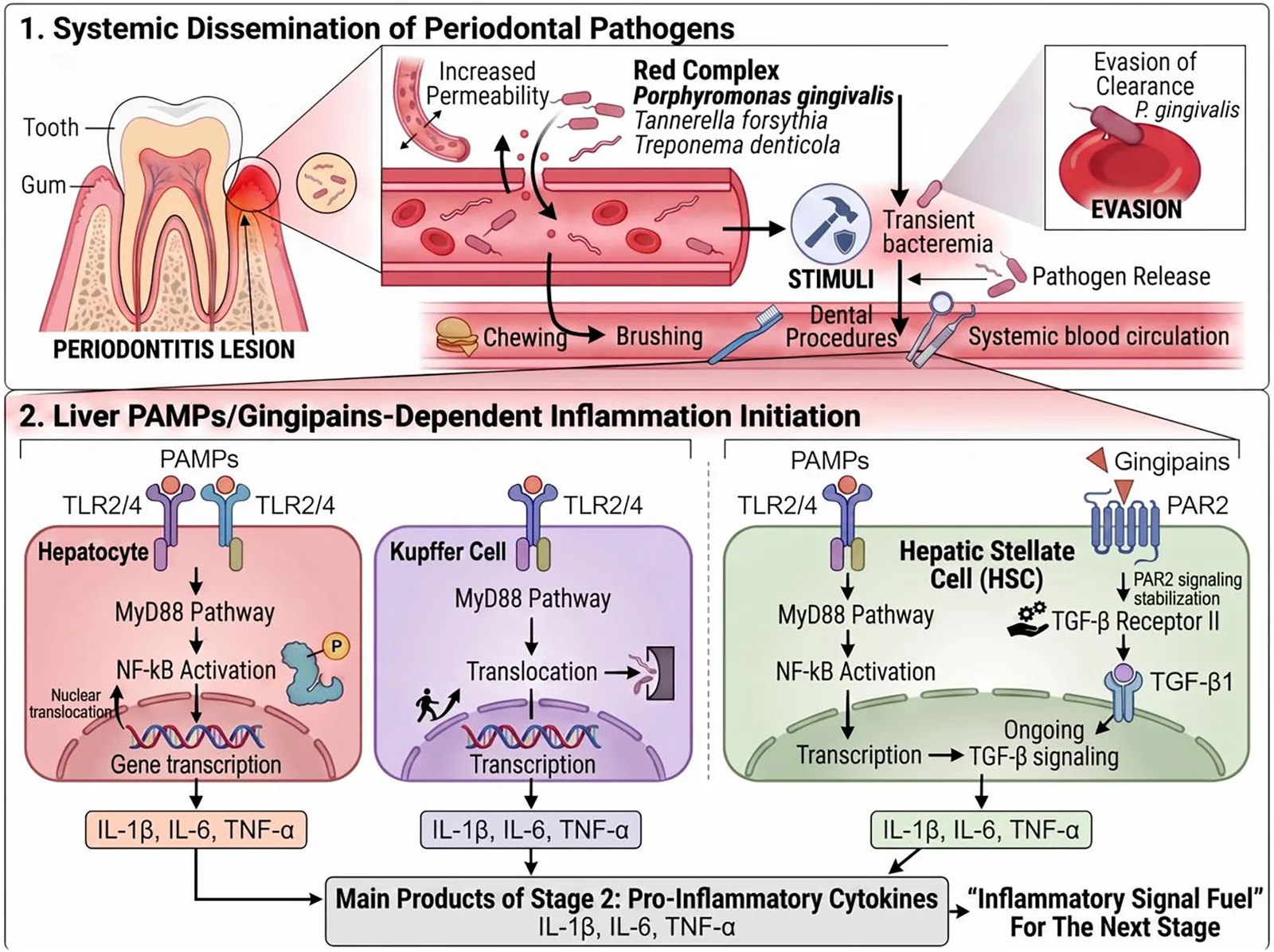
The initiation phase of the oral-gut-liver axis: periodontal pathogen dissemination and hepatic TLR/NF-κB activation. This schematic illustrates the initial events that bridge localized periodontal inflammation to hepatic fibrogenic signaling. In the periodontium, dysbiotic subgingival biofilms, particularly the "red complex" pathogens, disrupt the epithelial barrier. Periodontal pathogens and their virulence factors disseminate systemically via two primary routes: hematogenous spread through inflamed microvasculature and intestinal translocation following disruption of the gut microbiome. Upon reaching the liver, these pathogen-associated molecular patterns (PAMPs) activate Toll-like receptors 2 and 4 (TLR2/4) on Kupffer cells (KCs), hepatic stellate cells (HSCs), and hepatocytes. This triggers the MyD88-dependent pathway, leading to IκB-α phosphorylation and NF-κB nuclear translocation. The resulting transcriptional upregulation of pro-inflammatory genes represents the inflammatory output that concludes the initiation phase and primes the amplification loop.

### 3.1 Oral colonization and systemic spread of red complex pathogens

The core pathogenic microbial community of periodontal disease, particularly the “red complex,” represented by Porphyromonas gingivalis, Tannerella forsythia, and Treponema denticola, forms a highly synergistic biofilm within the periodontal pocket (Pretzl et al., 2017; Lafaurie et al., 2023). These Gram-negative anaerobes disrupt the periodontal tissue barrier by secreting virulence factors, thereby facilitating their dissemination from local foci into the systemic circulation (Widziolek et al., 2025). Clinical microbiological studies show that the detection rate and abundance of Red Complex bacteria in patients with severe periodontitis increase significantly with greater periodontal pocket depth and are particularly enriched at sites with probing depths ≥6 mm (Checchi et al., 2016; Nozawa et al., 2020). Primary routes of entry include increased microvascular permeability due to inflammation and transient bacteremia induced by chewing, toothbrushing, or dental procedures (Brun et al., 2020; Gualtero et al., 2023). P. gingivalis can bind to red blood cells to evade phagocytic clearance, enhancing systemic colonization (Damgaard et al., 2017). Direct evidence from animal studies confirms that P. gingivalis can migrate to the liver via the bloodstream in murine models (Ding et al., 2019; Gao et al., 2023). However, direct tracking of live periodontal pathogens from the oral cavity to the human liver has not been conclusively demonstrated, and the extent to which this hematogenous route contributes to hepatic pathology in humans remains unclear.

Beyond hematogenous spread, intestinal translocation—termed “gut-oralization” is increasingly recognized. In a mouse model, oral microbiota from patients with periodontitis, when transplanted into obese mice, have been shown to alter the gut microbiota, disrupt tight junctions, increase intestinal permeability, and promote LPS entry into the portal circulation (Wang et al., 2023). Although P. gingivalis may not be directly detected in the lower gut, it can expand IL-9^+^ CD4^+^ T cells in the small intestine, decrease microbial α-diversity, and dysregulate barrier-related genes (Sohn et al., 2022). Thus, both hematogenous and transintestinal routes represent plausible mechanisms, as hypothesized to complete the initiation phase based on preclinical observations, by delivering periodontal pathogen-associated molecular patterns (PAMPs) to the liver and gut.

### 3.2 Hepatic TLR/NF-κB activation: the inflammatory switch

*In vitro* and animal model studies have demonstrated that once periodontal pathogen-associated molecular patterns (PAMPs) reach the liver, they can activate Toll-like receptors 2 and 4 (TLR2/4) on hepatocytes, Kupffer cells (KCs), and hepatic stellate cells (HSCs) (Nagasaki et al., 2020). Whether this pathway operates identically in human livers *in vivo* remains to be fully established. P. gingivalis lipopolysaccharide (LPS) and lipoproteins bind to TLR2/4, triggering the MyD88-dependent pathway. In HepG2 hepatocytes, P. gingivalis LPS upregulates MyD88, increases phosphorylation of NF-κB p65 and JNK, and induces release of IL-1, IL-8, and TNF-α (Ding et al., 2019). Concurrently, gingipains activate protease-activated receptor 2 (PAR2) on HSCs, thereby stabilizing TGF-β receptor II and enhancing sensitivity to TGF-β1 (Nagasaki et al., 2020).PAR2 activation has been shown to promote hepatic stellate cell activation and collagen release in experimental liver fibrosis models (Knight et al., 2012). In animal models, periodontitis-induced liver injury is accompanied by elevated expression of TLR4, MyD88, and NF-κB (Yue et al., 2021). NF-κB serves as the core transcription factor downstream of TLR signaling. Upon IKK-mediated phosphorylation and degradation of IκB-α, NF-κB translocates to the nucleus and initiates transcription of pro-inflammatory genes, including IL-1β, IL-6, and TNF-α (Kiziltas, 2016; Shin et al., 2022). These cytokines not only amplify intrahepatic inflammation but also directly activate HSCs—TNF-α and IL-1β promote α-SMA and type I collagen expression in HSCs (Chen et al., 2021). In a mouse model of P. gingivalis infection combined with a high-fat diet, intrahepatic TNF-α and IL-6 were elevated, along with HSC activation markers and worsened fibrosis (Nagasaki et al., 2020). This NF-κB-driven inflammatory output, as demonstrated in preclinical models, may initiate the amplification phase by acting on distal organs, particularly the intestinal epithelium.

## 4 Amplification phase: intestinal barrier disruption and endotoxin storm

This phase is defined by the transition from a self-limiting inflammatory signal to a self-perpetuating fibrogenic loop. The pro-inflammatory cytokines released from the liver (TNF-α, IL-1β, IL-6) and bacterial components circulate systemically and act on the intestinal epithelium, downregulating tight junction proteins and depleting goblet cells, thereby compromising the physical and mucosal barriers. This barrier disruption triggers a “leaky gut,” allowing massive quantities of endotoxins and bacterial products to enter the portal circulation. Once in the liver, these endotoxins activate TLR4 signaling, further amplifying Kupffer cell M1 polarization and HSC activation. This establishes a positive feedback loop: hepatic inflammation is associated with intestinal barrier dysfunction, which in turn may contribute to hepatic inflammation. Beyond endotoxin translocation, periodontal dysbiosis also disrupts gut microbial metabolism of bile acids and other metabolites, creating parallel amplification pathways that reinforce the leaky gut phenotype and sustain hepatic inflammation (**Figure 2**).

**Figure 2:**
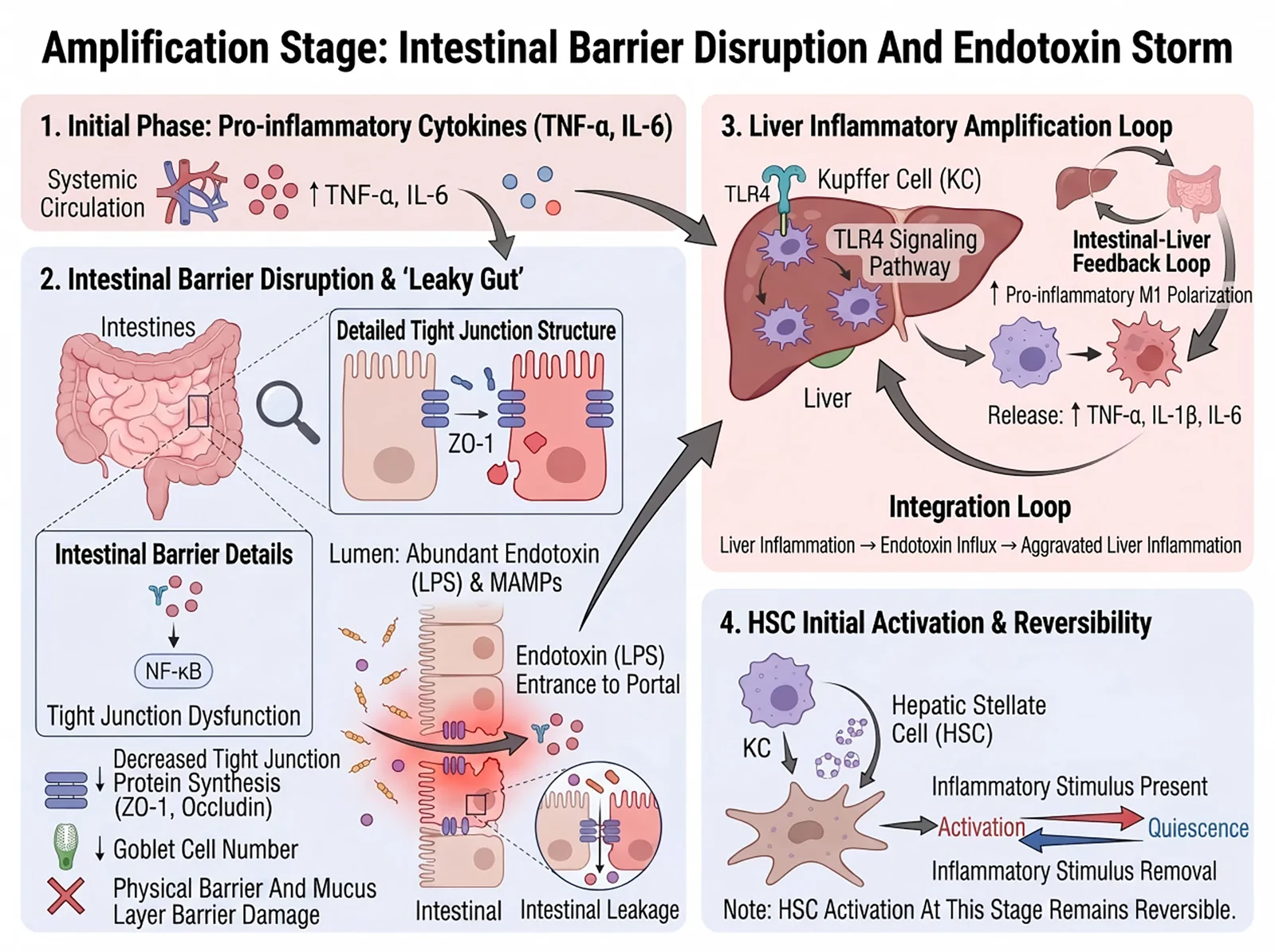
The amplification phase: intestinal barrier disruption, endotoxin translocation, and the self-perpetuating fibrogenic loop. This image depicts the transition from hepatic inflammation to a systemic self-amplifying loop driven by gut-liver crosstalk. Hepatic pro-inflammatory cytokines released during the initiation phase act on the intestinal epithelium. This systemic inflammation, coupled with periodontal dysbiosis, downregulates tight junction proteins, depletes goblet cells, and impairs the intestinal mucosal barrier. The resultant “leaky gut” permits massive translocation of luminal endotoxins (LPS) and other microbe-associated molecular patterns (MAMPs) into the portal circulation. This endotoxin overload reaches the liver, where it hyperactivates TLR4/NF-κB signaling in Kupffer cells and promotes polarization toward a pro-inflammatory M1 phenotype. M1-polarized KCs release additional inflammatory mediators, which in turn activate hepatic stellate cells (HSCs). This establishes a positive feedback loop: hepatic inflammation drives intestinal barrier dysfunction, which in turn fuels hepatic inflammation, thereby amplifying the fibrogenic signal. HSCs initially activate during this reversible phase.

### 4.1 Periodontal inflammation-induced downregulation of intestinal tight junctions

*In vivo* experiments demonstrate that in periodontitis-complicated liver disease models, colonic tight junction proteins ZO-1 and occludin are significantly downregulated, accompanied by elevated serum LPS and intestinal fatty acid-binding protein (I-FABP) levels (Feng et al., 2025; Jiacheng et al., 2025). Systemic inflammation activates NF-κB signaling in intestinal epithelial cells, thereby inhibiting tight junction synthesis (Li et al., 2022; Jiacheng et al., 2025). In animal models, periodontitis reduces ZO-1 and occludin expression in the ileum or colon and decreases goblet cell numbers (Zhang et al., 2019). This structural impairment transforms the intestinal barrier from a passive defense line into an active contributor to the fibrogenic loop.

### 4.2 Increased permeability is associated with endotoxin translocation and hepatic inflammatory amplification

Leaky gut allows luminal LPS and other microbe-associated molecular patterns (MAMPs) to cross the epithelium and enter the portal venous system, reaching the liver (Zhang et al., 2025). In patients with cirrhosis, intestinal permeability increases, and intestinal-origin bacterial DNA is detectable in serum, correlating with systemic inflammation (Tyc et al., 2025). Periodontitis or oral pathogen colonization exacerbates barrier damage, promotes LPS entry, and activates KC and HSC fibrosis via hepatic TLR4 signaling in animal models (Feng et al., 2025; Jiacheng et al., 2025). Antibiotic or barrier-protectant interventions reduce serum LPS levels and alleviate liver inflammation and fibrosis (Jiacheng et al., 2025). Direct evidence in humans linking periodontitis to increased gut permeability and subsequent hepatic TLR4 activation remains limited, as most studies rely on surrogate markers such as serum LPS or I-FABP rather than direct measurements of portal endotoxin levels or hepatic TLR4 activity.

The resultant endotoxin overload directly augments the hepatic inflammatory response, closing the first half of the amplification loop. Within the liver, this amplified LPS signal drives Kupffer cell polarization toward a pro-inflammatory M1 phenotype, creating a persistent inflammatory microenvironment. Periodontitis induces the accumulation of KCs, B cells, and Th17 cells in the liver, upregulating pro-inflammatory genes and activating NF-κB in B cells (Bai et al., 2022). Once activated by LPS or other PAMPs, Kupffer cells release pro-inflammatory cytokines and promote HSC activation through TLR4-dependent mechanisms and extracellular vesicles (Wang et al., 2025). In NASH models, monocyte-derived CD207^+^ macrophages expand and collaborate with KCs to maintain inflammation (Zhang et al., 2021). ANGPTL4, upregulated in cirrhosis, promotes KC polarization and the release of pro-inflammatory factors and enhances HSC activation via TLR4/NF-κB; interference with ANGPTL4 inhibits these processes (Zhang et al., 2021). Galectin-12 deficiency promotes KC M2 polarization, which upregulates TGF-β1 via STAT6, thereby indirectly promoting HSC myofibroblastic transformation and illustrating context-dependent effects (Lee et al., 2023). During this amplification phase, HSCs are initially activated by both direct inflammatory cytokines and amplified endotoxin signaling. This initial activation remains reversible if the inflammatory stimulus is removed, whereas the transition to a hypothesized irreversible, self-sustaining state defines the maintenance phase, although longitudinal human studies are needed to confirm this.

### 4.3 Feedback regulation between changes in gut microbiota composition and disruptions in secondary bile acid metabolism

Periodontal inflammation not only directly affects the intestinal barrier but also indirectly amplifies barrier dysfunction by altering gut microbiota composition and disrupting secondary bile acid metabolism. Gut dysbiosis is often characterized by reduced short-chain fatty acid (SCFA)-producing bacteria and an enrichment of opportunistic pathogens, which, in turn, affects bile acid deconjugation and transformation, leading to abnormal levels of secondary bile acids (Huang et al., 2025). However, the causal direction between bile acid alterations, gut dysbiosis, and periodontal disease in human MASLD patients remains unclear, as most human studies are cross-sectional and cannot distinguish cause from consequence.

Studies in animal models and cell culture have shown that disturbances in secondary bile acid metabolism can impair intestinal barrier function. For example, the primary bile acid chenodeoxycholic acid (CDCA) can reduce epithelial resistance and decrease occludin expression at high concentrations. In contrast, the secondary bile acid lithocholic acid (LCA) exerts a protective effect (Sarathy et al., 2017). In models of liver fibrosis, periodontal disease is often associated with reduced fecal levels of secondary bile acids, such as LCA and DCA, and decreased FXR/TGR5 signaling activity, further weakening the intestinal barrier and exacerbating liver inflammation (Zhao et al., 2024; Huang et al., 2025). Intervention studies have shown that targeted modulation of bile acid metabolism can restore tight junction protein expression, improve intestinal barrier function, and alleviate liver fibrosis (Gupta et al., 2020; Yang et al., 2025). Thus, the bile acid-microbiota axis constitutes a parallel amplification pathway, reinforcing the leaky gut phenotype through metabolic rather than purely inflammatory mechanisms.

### 4.4 Microbial metabolites as key mediators in the oral-gut-liver axis

Beyond the well-characterized roles of LPS and bacterial translocation, emerging evidence indicates that gut microbiota-derived metabolites—including short-chain fatty acids, indole derivatives, trimethylamine N-oxide (TMAO), and secondary bile acids—serve as critical signaling molecules that modulate hepatic inflammation and fibrogenesis (Lyu et al., 2025). Periodontal disease-induced gut dysbiosis alters the production of these metabolites, creating a metabolic environment that may protect against or exacerbate liver fibrosis (Simas et al., 2021).

Short-chain fatty acids, primarily acetate, propionate, and butyrate, are fermentation products of dietary fiber produced by gut commensals and play a generally protective role in liver health (Simas et al., 2021). SCFAs enhance intestinal barrier integrity by promoting tight junction protein expression, modulate immune responses via G protein-coupled receptors, and suppress hepatic inflammation through inhibition of NF-κB signaling (Simas et al., 2021). In the context of periodontal disease, P. gingivalis infection has been shown to reduce the abundance of SCFA-producing microbiota in murine models, which is associated with promoting hepatic steatosis and metabolic dysfunction. Reduced SCFA production has been identified as an important pathogenic factor in MASLD progression (Simas et al., 2021). However, whether SCFA depletion directly mediates the transition from steatosis to fibrosis in periodontitis-associated liver disease remains an active area of investigation.

Indole derivatives, including indole-3-propionic acid (IPA) and indole-3-acetic acid (IAA), are gut microbial metabolites of dietary tryptophan that exert pleiotropic effects on liver metabolism and fibrosis. IPA is lower in individuals with obesity and type 2 diabetes and exhibits hepatoprotective potential by reducing markers of hepatic stellate cell activation. Gut bacteria-produced indole activates the hepatic cytochrome P450 (CYP) eicosanoid pathway through pregnane X receptor-dependent mechanisms, thereby protecting the liver against injury and fibrosis (Zhang et al., 2025). Conversely, IPA can directly activate human hepatic stellate cells through ROS-induced JNK and p38 signaling pathways *in vitro*, suggesting that the role of tryptophan metabolites in liver fibrosis is context-dependent—potentially protective in the intact gut-liver axis but profibrotic under conditions of metabolic dysfunction or dysbiosis (Yuan et al., 2023). The aryl hydrocarbon receptor (AhR) has been identified as a central hub linking microbiota-derived indoles to hepatic metabolic reprogramming in MASLD. Whether periodontal dysbiosis alters the production of specific indole derivatives, and how these alterations influence hepatic fibrogenesis, warrants further investigation.

Trimethylamine N-oxide (TMAO) is a gut microbe-dependent metabolite generated from dietary precursors (choline, phosphatidylcholine, and L-carnitine) through sequential conversion by gut microbiota to trimethylamine (TMA) and subsequent hepatic oxidation by flavin-containing monooxygenase 3 (FMO3) (Jang et al., 2024). Chronic exposure to elevated TMAO has been associated with enhanced fibrosis propensity in multiple organ systems, including metabolic dysfunction-associated steatotic liver disease (Jang et al., 2024). *In vitro* studies have demonstrated that TMAO promotes lipid deposition and fibrosis in fatty liver cells. Mechanistically, TMAO may activate pro-inflammatory and pro-fibrotic signaling pathways, including TGF-β/Smad and NF-κB, although the precise molecular mechanisms remain incompletely defined. Periodontal disease, by inducing gut dysbiosis, may increase TMAO production by enriching TMA-producing bacterial taxa, thereby contributing to hepatic fibrogenesis (Lyu et al., 2025). However, direct evidence linking periodontitis-associated TMAO elevation to liver fibrosis in humans is currently limited.

Secondary bile acids, as discussed in Section 4.3, represent another critical class of microbial metabolites whose dysregulation links periodontal dysbiosis to hepatic fibrosis. Gut bacteria convert primary bile acids to secondary bile acids through deconjugation and dehydroxylation; periodontal disease-induced gut dysbiosis reduces bacterial bile salt hydrolase activity, thereby decreasing secondary bile acid production and impairing FXR/TGR5 signaling (Gupta et al., 2020; Huang et al., 2025). Secondary bile acids, such as lithocholic acid (LCA), can be hepatotoxic at high concentrations and have been implicated in the progression of liver fibrosis (Schnabl et al., 2025). The balance between protective and deleterious effects of bile acids is finely tuned and likely disrupted by periodontal inflammation.

Collectively, these microbial metabolites constitute a complex metabolic network through which periodontal dysbiosis may influence hepatic fibrogenesis. While evidence for SCFAs, indoles, TMAO, and secondary bile acids is rapidly accumulating, most data come from animal models or *in vitro* studies, and direct human evidence—particularly linking periodontitis-specific dysbiosis to metabolite alterations and subsequent fibrosis—remains limited. Future research should integrate metabolomic profiling with longitudinal cohort studies to clarify causal relationships and identify metabolite-based biomarkers or therapeutic targets for periodontal disease-associated liver fibrosis.

## 5 Maintenance phase: liver-intrinsic mechanisms sustaining fibrosis

The maintenance phase represents the transition from inflammation-driven, reversible HSC activation to a self-sustaining fibrotic state that persists even after the initial oral trigger subsides. This phase is governed by liver-intrinsic mechanisms that are hypothesized to lock the fibrotic program in place, including HSC autocrine signaling, epigenetic reprogramming, extracellular matrix (ECM) crosslinking, liver sinusoidal endothelial cell (LSEC) capillarization, and bile acid–FXR/TGR5 dysregulation. These mechanisms create a pro-fibrotic microenvironment that is no longer dependent on continuous external inflammatory inputs (**Figure 3**).

**Figure 3:**
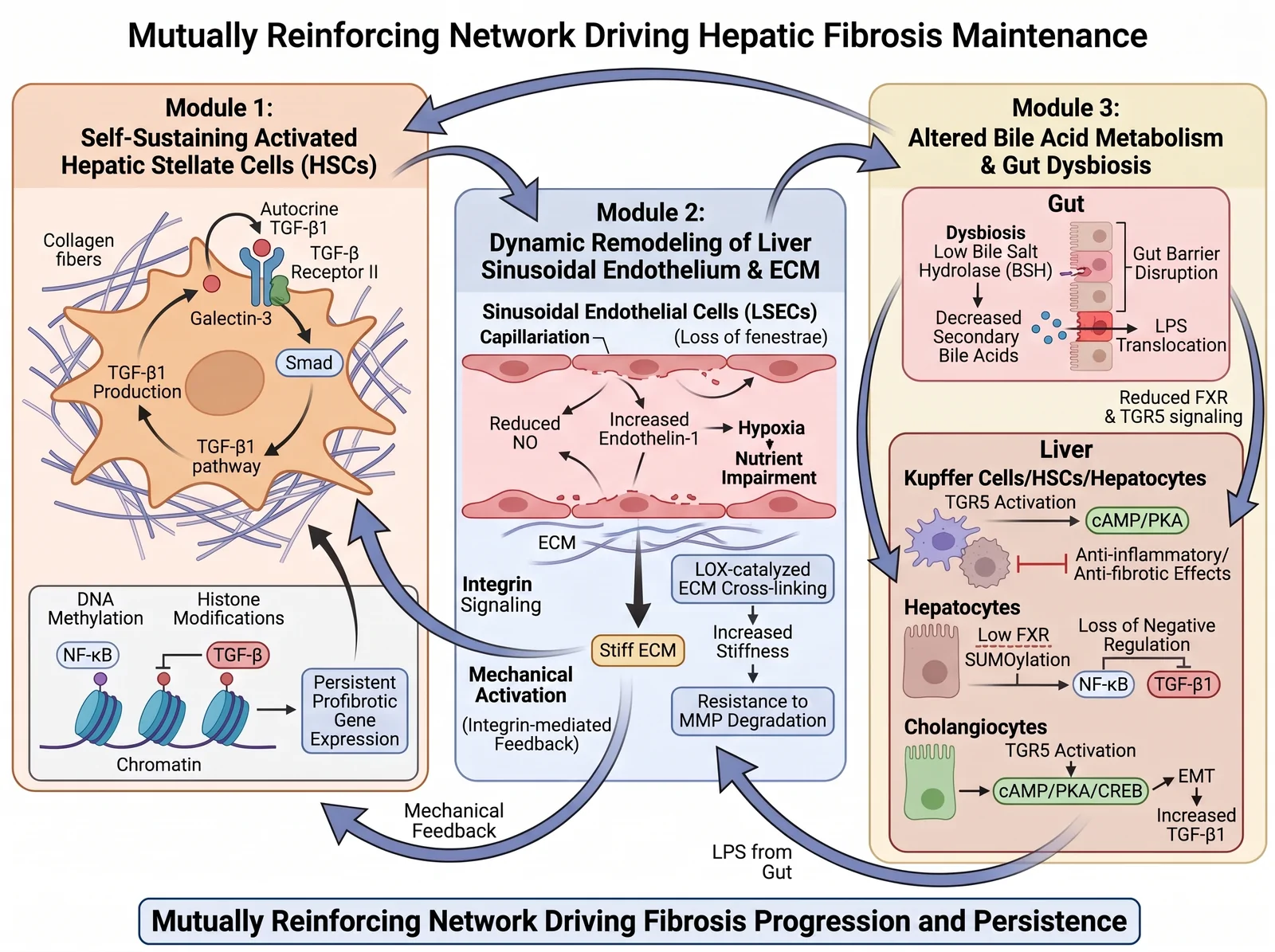
Maintenance phase of liver fibrosis: liver-intrinsic mechanisms sustaining HSC activation and fibrotic progression. This schematic summarizes the key self_perpetuating pathways within the liver that render fibrosis irreversible even after the initial periodontal trigger subsides. Activated hepatic stellate cells (HSCs) establish an autocrine positive feedback loop by secreting TGF-β1, which signals via Smad to sustain their own activation and collagen production; galectin-3 released from HSCs stabilizes TGF-β receptor II, further amplifying TGF-β sensitivity. Chronic inflammatory stimuli induce persistent epigenetic reprogramming, including DNA methylation and histone modifications at pro_fibrotic gene promoters, creating “molecular scars” that lock HSCs in an activated state. Liver sinusoidal endothelial cells (LSECs) undergo capillarization—loss of fenestrae and formation of a continuous basement membrane—leading to reduced nitric oxide (NO), increased endothelin-1, exacerbated hypoxia, and portal hypertension. Deposited extracellular matrix (ECM) is crosslinked by lysyl oxidase (LOX), increasing stiffness and resistance to matrix metalloproteinase (MMP) degradation; this stiffened ECM physically impedes repair and, via integrin signaling, further activates HSCs, forming a mechanical positive feedback loop. Altered bile acid pool, with decreased secondary bile acids due to gut dysbiosis, impairs FXR and TGR5 signaling. SUMOylation further suppresses FXR activity, relieving its inhibition of NF-κB and TGF-β1. TGR5 exhibits cell_type_dependent effects: in Kupffer cells, hepatocytes, and HSCs, TGR5 activation promotes anti_inflammatory/anti_fibrotic responses via cAMP/PKA. In cholangiocytes, it stimulates the cAMP/PKA/CREB axis, inducing epithelial_mesenchymal transition (EMT) and TGF-β1 expression, thus worsening fibrosis. These interconnected loops collectively maintain the fibrotic phenotype and drive disease progression.

### 5.1 Core maintenance driver: HSC autocrine TGF-β signaling and epigenetic reprogramming

HSCs can sustain their own activation through autocrine positive feedback loops. Activated HSCs produce large amounts of TGF-β1, which, through Smad signaling, continuously stimulates HSC activation and collagen production, independent of exogenous stimuli (Tahashi et al., 2002). Furthermore, galectin-3 released from HSCs stabilizes TGF-β receptor II, increasing cellular sensitivity to TGF-β1 and amplifying this autocrine loop (Henderson et al., 2006). Chronic inflammatory exposure induces epigenetic reprogramming of HSCs. Sustained NF-κB and TGF-β signaling lead to persistent changes in DNA methylation patterns and histone modifications at the promoters of pro-fibrotic genes (Teng and Ghoshal, 2015; Li et al., 2023). These ‘molecular scars’ are thought to lock HSCs in an activated state, based largely on *in vitro* and animal studies. Even after the initial LPS or cytokine stimulus is withdrawn, the epigenetic landscape maintains the fibrotic transcriptional program, which is a key reason advanced fibrosis is difficult to reverse (Zhang et al., 2016; Li et al., 2023).

### 5.2 Microenvironmental reinforcement: LSEC capillarization and ECM crosslinking

The fibrotic microenvironment itself drives maintenance. Liver sinusoidal endothelial cells (LSECs) under sustained inflammatory and oxidative stress undergo capillarization—loss of fenestrae and formation of a continuous basement membrane (Wang and Liu, 2021; Wang et al., 2021). This capillarization impairs exchange of substances between sinusoidal blood and hepatocytes, exacerbating hypoxia and nutrient deprivation and further promoting HSC activation. Additionally, capillarized LSECs produce less nitric oxide (NO) and more endothelin-1, thereby increasing intrahepatic resistance and contributing to portal hypertension (Wang et al., 2021). The deposited ECM is not only excessive but also qualitatively altered. Enzymes such as lysyl oxidase (LOX) catalyze collagen crosslinking, making the ECM highly rigid and resistant to degradation by matrix metalloproteinases (MMPs) (Li et al., 2023). This crosslinked ECM forms a physical barrier that impairs liver function and hinders the infiltration of repair cells and therapeutic agents, creating a self-perpetuating fibrotic niche. Mechanical stiffness further activates HSCs via integrin-mediated signaling, thereby closing another positive feedback loop (Li et al., 2023).

### 5.3 Metabolic maintenance: bile acid–FXR/TGR5 dysregulation

Gut microbes generate secondary bile acids exclusively: the liver synthesizes primary bile acids, conjugates them, and secretes them into the intestine, where gut bacteria deconjugate and dehydroxylate them to form secondary bile acids such as lithocholic acid (LCA) and deoxycholic acid (DCA) (Schnabl et al., 2025). The bile acid pool composition therefore depends heavily on gut microbial community structure. Periodontal disease-induced gut dysbiosis alters this metabolic conversion, reducing protective secondary bile acids and increasing the primary-to-secondary bile acid ratio, which may contribute to hepatic inflammation and fibrosis through impaired FXR/TGR5 signaling (Gupta et al., 2020; Huang et al., 2025).

Bile acids (BAs) are important signaling molecules regulating hepatic metabolism, inflammation, and fibrosis through the nuclear receptor FXR and the membrane receptor TGR5. In MASLD/MASH, the bile acid pool composition is altered, with decreased secondary bile acids, leading to weakened FXR and TGR5 signaling and reduced anti-inflammatory protection (Gillard et al., 2022). FXR activation suppresses HSC activation by inhibiting NF-κB and downregulating pro-fibrotic mediators such as TGF-β1 (Gao et al., 2018). However, in activated HSCs, SUMOylation impairs FXR signaling, limiting its antifibrotic efficacy (Zhou et al., 2020). TGR5 exerts anti-inflammatory effects in Kupffer cells via the cAMP/PKA/CREB pathway, but its role is context-dependent. In cholestatic models, TGR5 activation in cholangiocytes promotes EMT and TGF-β1 expression, exacerbating fibrosis (Tang et al., 2026). In metabolic liver disease, TGR5 deficiency worsens steatosis but reduces fibrosis markers in some models, owing to compensatory changes in serotonin (Spatz et al., 2021). Bile acid dysregulation and gut dysbiosis form a reciprocal loop. Periodontal disease-induced gut dysbiosis has been proposed to reduce bacterial bile salt hydrolase activity, based on indirect evidence from animal models and observational human microbiome studies, thereby potentially decreasing secondary bile acid production, which may impair FXR/TGR5 signaling, further compromising intestinal barrier function and promoting continued LPS translocation, although direct human evidence for this sequence is currently limited (Gupta et al., 2020; Huang et al., 2025). This metabolic dysregulation is hypothesized to sustain hepatic inflammation and HSC activation even after the oral infection is controlled, making the bile acid axis a potential key maintenance node that warrants further investigation in human studies.

Importantly, the role of TGR5 in liver fibrosis is cell-type and etiology-dependent—a nuance with direct therapeutic implications. In Kupffer cells and hepatocytes, TGR5 activation suppresses NF-κB and NLRP3 inflammasome signaling via the cAMP/PKA pathway, exerting anti-inflammatory and anti-fibrotic effects (Xiang et al., 2026). In hepatic stellate cells, TGR5 agonism inhibits activation and mitochondrial fission, further supporting an anti-fibrotic profile (Keitel and Häussinger, 2018; Ma et al., 2021). However, in cholangiocytes, TGR5 activation stimulates the cAMP/PKA/CREB axis, promoting epithelial-mesenchymal transition and TGF-β1-driven fibrosis (Tang et al., 2026). This pro-fibrotic effect has been documented in hepatolithiasis and cholestatic models, suggesting that TGR5 agonists may benefit metabolic liver diseases but may be deleterious in biliary pathologies (Tang et al., 2026). Therefore, rather than a universal “pro-fibrotic” or “anti-fibrotic” designation, TGR5 should be viewed as a context-dependent modulator whose therapeutic targeting must be guided by the underlying liver disease etiology—favoring TGR5 agonism in MASLD/MASH but exercising caution in cholestatic or biliary-predominant fibrotic conditions. This distinction is particularly relevant to the oral-gut-liver axis, where periodontal disease-associated fibrosis most commonly arises in a metabolic context, in which TGR5 agonism would be predicted to confer net benefit.

## 6 Current challenges and future directions

While the mechanistic pathways described above provide a coherent biological framework, the clinical evidence remains largely correlative. Definitive demonstration of causality will require prospective interventional studies with hard histologic endpoints. Nevertheless, the existing evidence is compelling enough to warrant further investigation and support interdisciplinary collaboration between dentists and hepatologists. Despite the substantial progress reviewed above in elucidating the oral-gut-liver axis as a mechanistic bridge between periodontal disease and liver fibrosis, translating these insights into clinical practice faces three interconnected categories of challenges: technical bottlenecks in mechanistic tracing, limitations in causal inference, and gaps in interventional evidence. This section synthesizes these challenges and proposes a strategic roadmap for future research.

### 6.1 Distinguishing evidence levels in mechanistic pathways

The three-phase framework proposed in this review synthesizes these evidence levels and should be regarded as a heuristic model rather than a proven pathogenic sequence. The evidence tiers presented below are intended to help readers assess the strength of the underlying data for each mechanistic step. A recurring challenge in synthesizing the oral-gut-liver axis literature is the variable quality and strength of the evidence supporting each proposed mechanism. Based on the available data, the mechanistic pathways reviewed in this article can be stratified into three broad categories by current evidence level. The strongest evidence—derived from human observational studies with consistent replication and supported by animal models—supports the epidemiological association between periodontal disease severity and liver fibrosis stage, as well as elevated systemic inflammatory markers and endotoxemia in patients with periodontitis and liver disease. Moderately supported mechanisms-–demonstrated in animal models or *in vitro* and consistent with human biomarker data-–include TLR/NF-κB-mediated inflammatory responses to periodontal PAMPs, intestinal tight junction protein downregulation, and cytokine-driven HSC activation. The most speculative mechanisms—which remain to be directly tested in relevant human or animal models—include the precise sequence of events linking periodontal dysbiosis to specific alterations in the bile acid pool, the direct tracking of live periodontal pathogens from the oral cavity to the human liver, and the causal role of epigenetic reprogramming in sustaining fibrosis specifically in the context of periodontal disease. We have explicitly indicated these varying levels of evidence throughout the manuscript by using language that reflects the strength and nature of the underlying data, and we highlight these distinctions in the discussions above to guide future validation studies. The most speculative mechanisms—which remain to be directly tested in relevant human or animal models—include the precise sequence of events linking periodontal dysbiosis to specific alterations in the bile acid pool, the direct tracking of live periodontal pathogens from the oral cavity to the human liver, the causal role of epigenetic reprogramming in sustaining fibrosis specifically in the context of periodontal disease, and the specific contributions of individual microbial metabolites to periodontal disease-associated hepatic fibrogenesis in humans.

### 6.2 The critical evidence gap: absence of RCTs with histological reversal as a hard endpoint

Among the three categories of challenges discussed in this section—technical bottlenecks, confounding in observational studies, and lack of interventional trials—the absence of high-quality randomized controlled trials (RCTs) with liver histological reversal as a primary endpoint constitutes the most critical barrier to clinical translation. Without such trials, periodontal treatment cannot be recommended as a standard adjunctive therapy for liver fibrosis, regardless of the mechanistic plausibility or the strength of observational associations.

Epidemiological studies have consistently revealed associations between periodontal disease severity and liver fibrosis stage (summarized in **Table 1**). Most evidence derives from cross-sectional or observational cohort designs that cannot establish causality (Åberg and Helenius-Hietala, 2022; Liu et al., 2026). Confounding remains a persistent threat. Factors such as age, obesity, diabetes, alcohol intake, smoking, dietary patterns, and socioeconomic status may influence both periodontitis and liver fibrosis, thereby undermining the specificity of the observed associations. Large cohorts such as the Finnish Health 2000 study and the UK Biobank have applied multivariable adjustments. Residual confounding cannot be entirely excluded. Many of these confounding factors influence the gut microbiota and intestinal barrier function independently of periodontal status (Helenius-Hietala et al., 2019; Liu et al., 2026; Ye et al., 2026). Of the three categories of challenges discussed here—confounding, lack of RCTs, and unclear dose-response—residual confounding is arguably the most intractable, as statistical adjustment alone cannot fully address it. This suggests that future causal inference should shift from purely observational designs to genetically informed approaches, such as Mendelian randomization, which are less susceptible to confounding by lifestyle factors.

Beyond confounding, the interventional evidence base remains critically underdeveloped. The absence of histological-endpoint RCTs is the most critical evidence gap in the field and directly undermines the clinical credibility of the periodontal-liver axis hypothesis. To date, no high-quality, large-sample, multicenter randomized controlled trial has evaluated liver histological reversal as a primary endpoint following periodontal therapy. Most existing intervention studies have assessed surrogate markers such as serum ALT, non-invasive fibrosis scores, or systemic inflammatory cytokines (Kamata et al., 2022). While these markers may reflect liver inflammation or fibrosis risk, they cannot be equated with improvements in fibrosis staging as defined by liver biopsy. Animal studies consistently show that periodontal intervention reduces hepatic collagen deposition and α-SMA expression (Mester et al., 2019; Xie et al., 2022). These findings have not been translated into human histological evidence. Furthermore, several studies reporting reductions in systemic inflammatory markers did not concurrently evaluate hepatic histopathology, precluding any causal attribution of observed biomarker changes to improved liver fibrosis (Bajaj et al., 2018; Krasniqi et al., 2025).

This gap should therefore be prioritized over other research directions in the clinical translational agenda. Future RCTs should be designed with adequate statistical power, based on clinically meaningful effect sizes—for example, the 12 U/L average ALT reduction observed in the SRP group in a recent multicenter trial—and should incorporate liver biopsy or at least validated non-invasive fibrosis assessment tools as primary or co-primary endpoints (Gheorghe et al., 2021; Kamata et al., 2022; Tamaki et al., 2022). Intervention protocols must be strictly standardized, clearly defining the extent, frequency, and types of instruments used, as well as standardized adjunctive therapy protocols, to minimize performance bias. Follow-up durations should span a sufficient biological window to capture meaningful changes in fibrotic tissue remodeling.

Host genetic variation adds another layer of complexity, particularly TLR4 polymorphisms, which alter LPS responsiveness and have been associated with accelerated fibrosis in chronic hepatitis C but protective effects in NAFLD. Whether these polymorphisms modify the periodontal disease-liver fibrosis association remains unexplored. Addressing this question will require adequately powered genetic association studies that stratify patients by both periodontal status and TLR4 genotype, as well as mechanistic studies using patient-derived induced pluripotent stem cell (iPSC)-differentiated hepatocytes or macrophages with defined TLR4 haplotypes. Such genetic approaches could simultaneously address confounding and causal inference. Therefore, we argue that the highest priority for future clinical research should be a rigorously designed, multicenter RCT that uses liver biopsy or validated non-invasive surrogates as co-primary endpoints, with adequate statistical power and follow-up duration to capture meaningful fibrotic remodeling.

### 6.3 Technical bottlenecks: microbial tracing, multi-omics, and advanced models

Accurately tracing the translocation of oral pathogens—particularly low-abundance red complex species—across the oral, gut, and hepatic compartments remains a formidable technical hurdle. Current 16S rRNA gene sequencing offers limited resolution at the species or strain level, making it difficult to distinguish highly homologous commensals from pathogenic strains and virtually impossible to track specific lineages across anatomical niches (Oikonomou et al., 2018). This fundamental limitation undermines definitive source tracking of oral pathogens to the liver, and without this resolution, downstream mechanistic studies risk investigating the wrong microbial targets. While metagenomic shotgun sequencing improves taxonomic granularity, host DNA contamination in low-biomass samples often confounds it, and standardized bioinformatics workflows for cross-niche source tracking remain lacking (Tilg et al., 2022). Liver tissue sampling, moreover, is invasive and impractical for longitudinal monitoring, thereby forcing most studies to rely on indirect inferences from fecal or peripheral blood samples—approaches susceptible to contamination and artifacts of transient bacteremia.

Additionally, the choice of microbial profiling technology imposes inherent limitations that require careful consideration. Metatranscriptomics, which measures the active transcriptional activity of gut microbial communities rather than mere taxonomic presence, provides a more functional view of the oral-gut-liver axis. However, it faces substantial challenges, including RNA instability, rapid degradation during sample processing, and the difficulty of depleting abundant host RNA in tissue or blood samples, which can obscure microbial signals. Furthermore, metatranscriptomic analysis is highly sensitive to sequencing depth and often requires large computational resources for accurate read mapping, particularly when distinguishing highly homologous sequences between closely related species. Metabolomics offers a direct readout of microbial functional output and host-microbiome crosstalk. Still, its application to the oral-gut-liver axis is constrained by metabolite chemical diversity, limited spectral library coverage for identifying unknown compounds, and the difficulty of assigning specific metabolites to microbial or host origin without stable isotope tracing. Most human metabolomic studies in this field have been cross-sectional, limiting causal inference regarding the relationship between specific metabolites and fibrosis progression.

More recently, spatial transcriptomics and single-cell sequencing have emerged as powerful tools to dissect tissue-level heterogeneity in the oral-gut-liver axis. Spatial transcriptomics maps gene expression within the spatial architecture of liver tissue, potentially identifying fibrotic niches and their surrounding cellular ecosystems, while single-cell RNA sequencing deconvolutes cell-type-specific responses to periodontal-derived stimuli, such as activation states of individual hepatic stellate cells or Kupffer cells. However, applying these technologies to periodontal disease-associated liver fibrosis remains in its infancy. Their integration with multi-omics datasets is technically challenging, requiring sophisticated bioinformatics pipelines and substantial computational infrastructure. Moreover, the high cost of spatial and single-cell technologies limits their use in large-cohort studies, and careful study design is essential to ensure adequate statistical power to detect meaningful biological signals.

To overcome these limitations, future mechanistic investigations should prioritize developing highly sensitive, strain-resolving tracking strategies that integrate multiple orthogonal data types. These may include cultivation-independent strain-specific marker gene panels, metabolic fingerprinting, and host immune response signatures as proxies for prior exposure. Among these technical hurdles, the lack of strain-level resolution in 16S rRNA sequencing represents the most fundamental limitation. Future efforts should prioritize developing strain-resolving technologies as a prerequisite for mechanistic validation, rather than pursuing multi-omics integration in parallel without a clear hierarchical framework. Additionally, the application of organoid-based co-culture systems—combining human intestinal epithelial organoids with hepatic stellate cells or hepatocyte spheroids—offers a controlled *in vitro* platform to dissect the direct effects of translocated pathogens and their outer membrane vesicles (OMVs) on hepatic fibrogenic responses, circumventing the confounding variables inherent in animal models (Li et al., 2026; Zhao et al., 2026). Humanized gnotobiotic mice, colonized with patient-derived oral or fecal microbiota, provide a complementary *in vivo* platform that more faithfully recapitulates species-specific host-microbe interactions (Zhao et al., 2026). These advanced model systems are not interchangeable but complementary. Organoids offer high-throughput mechanistic screening with human cellular contexts, whereas humanized mice provide whole-organism physiological integration. Future research should adopt a “tiered” approach: initial mechanistic hypotheses generated in organoid co-cultures should be subsequently validated in humanized mouse models before proceeding to human trials. This sequential strategy would optimize resource allocation and increase the translational yield of preclinical findings. Ultimately, integrating multi-omics discovery, including metagenomic, metabolomic, transcriptomic, and proteomic data—with functional validation in these advanced model systems will accelerate the transition from correlation-based hypotheses to mechanistically validated pathways (Liu et al., 2026; Zhao et al., 2026).

### 6.4 Unresolved questions: intervention timing, dose-response, and patient stratification

A third translational gap concerns the unclear dose-response relationship between the timing, frequency, and intensity of periodontal treatment and hepatic benefits. The U.S. Veterans Compensation Cirrhosis Cohort Study reported that regular preventive dental care was associated with a dose-dependent reduction in the risk of decompensation and hepatocellular carcinoma (Bajaj et al., 2026). However, observational data cannot determine the optimal treatment frequency, nor can they distinguish whether early intervention yields greater benefits than late-stage treatment. Heterogeneous intervention protocols across studies—ranging from single-episode scaling and root planing (SRP) to SRP combined with systemic antibiotics or probiotics—further limit comparability and preclude the formulation of evidence-based clinical guidelines (Gheorghe et al., 2021; Kamata et al., 2022). Critically, no study has yet defined an “effective intervention window” based on baseline fibrosis stage, leaving clinicians uncertain whether periodontal treatment should be offered to all liver disease patients or specifically targeted to those with early-stage fibrosis. The heterogeneity in treatment protocols across studies reflects not merely methodological variation but a deeper uncertainty about the biological determinants of treatment response. The optimal intervention window and frequency likely vary by the stage of liver fibrosis and the functional status of the intestinal barrier—parameters prior studies have not systematically captured. The association between periodontitis and liver fibrosis is more pronounced in patients with MASLD or MASH, particularly among those with obesity and insulin resistance. Future trials should therefore prioritize enrollment of patients with biopsy-proven or non-invasively staged MASLD/MASH who also have moderate-to-severe periodontitis. The timing of intervention requires optimization: animal models demonstrate that periodontitis exacerbates high-fat diet-induced liver fibrosis, suggesting that early periodontal treatment—initiated at the stage of steatosis may interrupt the inflammatory cascade before fibrotic remodeling becomes established (Tari et al., 2026). Combination strategies, such as SRP plus sodium-glucose cotransporter 2 (SGLT2) inhibitors or nutritional interventions, should also be explored to improve both metabolic and fibrotic outcomes synergistically. Given the molecular heterogeneity of MASLD-associated liver fibrosis, with distinct subtypes characterized by pro-fibrotic macrophage signatures and differential expression of fibrosis-related genes such as COL1A2 and EFEMP2 (Pantano et al., 2021; He et al., 2023), future trials should incorporate baseline multi-omics profiling to stratify participants. Integration of host genomic, transcriptomic, and epigenomic data with oral and gut metagenomic profiles can yield multidimensional biomarker panels that identify “high-response” subgroups most likely to benefit from periodontal interventions (Lv et al., 2026; Wu et al., 2026). Pay attention to OMV load, systemic LPS levels, and the relative abundance of *P. gingivalis*, as these may predict treatment response. Thus, future efforts to define dose-response relationships should be embedded within stratified trial designs that account for baseline fibrosis stage and gut permeability, rather than treating all patients as a homogeneous population. Furthermore, host genetics adds complexity to patient stratification. Single-nucleotide polymorphisms (SNPs) in the human TLR4 gene—most notably TLR4 D299G and T399I—have been shown to alter receptor structure and attenuate LPS responsiveness, with estimated population frequencies of 6–10% in European populations and 2–4% in Asian populations. These polymorphisms have been associated with a reduced risk of certain inflammatory diseases but, paradoxically, with increased susceptibility to others, reflecting TLR4’s dual role in both host defense and inflammatory pathology. In the context of liver disease, the D299G polymorphism has been linked to accelerated fibrosis progression in chronic hepatitis C and to increased risk of cirrhosis in alcoholic liver disease—possibly due to impaired clearance of bacterial products and compensatory upregulation of alternative inflammatory pathways. However, studies have also reported protective associations with MASLD severity, suggesting that the net effect of TLR4 hyporesponsiveness depends on the underlying etiology and the relative contribution of LPS-driven versus sterile inflammation. Crucially, whether TLR4 polymorphisms modify the periodontal disease-liver fibrosis association has not been directly investigated. One could hypothesize that individuals with hyporesponsive TLR4 variants might be partially protected from the hepatic effects of periodontal-derived endotoxins—or that they might compensate by upregulating TLR2, thereby remaining susceptible via a different route. Addressing this question will require adequately powered genetic association studies that stratify patients by both periodontal status and TLR4 genotype, alongside mechanistic studies using patient-derived induced pluripotent stem cell (iPSC)-differentiated hepatocytes or macrophages with defined TLR4 haplotypes.

### 6.5 Establishing interdisciplinary oral-liver follow-up cohorts

The successful implementation of the above priorities—RCTs with mechanistic nesting, precision stratification, and combination strategies—ultimately depends on a robust research infrastructure. We therefore advocate establishing long-term, prospective, multicenter joint oral-liver cohorts as a foundational platform. The current fragmentation of evidence arises in part from disciplinary silos between dentistry and hepatology. We advocate establishing long-term, prospective, multicenter joint oral-liver cohorts that systematically collect standardized data from both domains. Oral parameters should include the Community Periodontal Index (CPI), clinical attachment loss (CAL), P. gingivalis-specific IgG titers, and salivary microbiome profiles; hepatic parameters should encompass Fibrosis-4 index (FIB-4), NFS, liver stiffness measurement, magnetic resonance imaging-proton density fat fraction (MRI-PDFF), and emerging serum biomarkers such as YKL-40 and Mac-2 binding protein glycosylation isomer (Kumagai et al., 2016; Jang et al., 2021). Drawing on the successful model of the South Korean National Health Insurance Database (n=165,032), which tracked the dose-response relationship between periodontal treatment frequency and MASLD risk (Shin et al., 2022; Wang et al., 2026), such platforms can validate causal relationships while also promoting clinical pathway integration—including routine periodontal screening in MASLD management and reciprocal hepatology referral from dental clinics. Ultimately, these interdisciplinary collaborations will accelerate the public health translation of “oral-liver axis” interventions. Such platforms would not only validate causal relationships but also promote clinical pathway integration—including routine periodontal screening in MASLD management and reciprocal hepatology referral from dental clinics—ultimately accelerating the public health translation of oral-liver axis interventions.

### 6.6 Potential phase-guided therapeutic opportunities: translational implications of the three-phase framework

A central premise of the three-phase framework—and a key distinction from previous non-temporal models of the oral-gut-liver axis—is that each phase of the fibrogenic cascade is dominated by distinct molecular drivers, which in theory makes each phase susceptible to distinct intervention strategies that could be tested in future studies. Whereas previous frameworks have described a sequential pathway from oral dysbiosis to hepatic fibrosis without dissecting the temporal evolution of the fibrogenic process, our stratification explicitly distinguishes the initial inflammatory trigger, the self-amplifying gut-liver inflammatory loop, and the liver-intrinsic maintenance phase. This phase-specific logic provides a rational basis for designing multi-node therapeutic protocols that move beyond the current “one-size-fits-all” approach to periodontal-liver intervention. However, the interventions discussed below are at markedly different stages of translational readiness. To maintain consistency with the evidence-tiering approach applied throughout this review (Section 6.1), we explicitly stratify these strategies into three maturity levels: (I) clinically translatable—agents with approved indications or in advanced-phase trials for related fibrotic conditions;(II) preclinically validated—mechanisms demonstrated in animal models but lacking human proof-of-concept data;(III) conceptual/proof-of-mechanism—strategies supported by *in vitro* studies or mechanistic inference, requiring substantial preclinical development before clinical consideration.

Initiation phase: targeting periodontal pathogen dissemination. The priority at this stage is to prevent or minimize the systemic spread of periodontal pathogens and their virulence factors. Strategies conceptually targeting this phase include anti-P. gingivalis vaccines, topical antimicrobial photodynamic therapy, and rigorous scaling and root planing (SRP) to reduce subgingival biofilm burden. Among these, SRP is the only intervention with established clinical feasibility in patients with liver disease, with observational studies reporting improvements in MELD scores and systemic inflammatory markers following periodontal therapy (Bajaj et al., 2018). Preclinical studies have shown that passive immunization against gingipains reduces soft tissue inflammation and bacterial dissemination in animal models, although human data remain preliminary (Shin et al., 2022). The vaccine approach should therefore be classified as maturity level III. In contrast, SRP—while supported by clinical observational data—lacks RCT-level evidence for hepatic histological benefit and thus sits between levels I and II. In practical terms, SRP can be recommended as part of comprehensive dental care for patients with chronic liver disease. Still, it cannot yet be endorsed as a specific anti-fibrotic therapy. Anti-P. gingivalis vaccines and photodynamic therapy remain experimental and should not be considered for clinical application outside well-designed trials.

Amplification phase: interrupting the gut–liver inflammatory loop. During the amplification phase, the therapeutic logic shifts to breaking the self-perpetuating inflammatory loop at the gut–liver interface. Candidate strategies include intestinal barrier protectants, TLR4 antagonists, and MyD88 inhibitors that block hepatic endotoxin sensing. In animal models of periodontitis-associated liver fibrosis, MyD88 inhibitors (e.g., TJ-M2010-5) and sodium butyrate have been shown to reduce serum LPS, restore occludin and ZO-1 expression, and attenuate hepatic collagen deposition (Zhang et al., 2021; Xie et al., 2022; Wang et al., 2026). However, no TLR4 antagonist or MyD88 inhibitor has yet been approved for fibrosis indications, and concerns about immunosuppression and infection further hinder clinical translation. These agents should therefore be classified as maturity level II (preclinically validated). Barrier protectants such as probiotics, prebiotics, and short-chain fatty acid supplementation have entered early-phase human trials in metabolic liver disease. Still, their specific efficacy in periodontitis-associated fibrosis remains unproven. This category thus occupies an intermediate position between levels II and I—promising but requiring dedicated RCTs with histological or validated non-invasive endpoints.

Maintenance phase: reversing or halting established fibrosis. Once HSC activation has become autonomous through autocrine TGF-β signaling, epigenetic reprogramming, and ECM crosslinking, the therapeutic goal shifts to reversing or halting established fibrosis. Among the strategies conceptually relevant to this phase, FXR agonists, particularly obeticholic acid (OCA), have advanced the furthest in clinical development. OCA has been evaluated in Phase III trials for non-alcoholic steatohepatitis (NASH) with fibrosis, demonstrating histological improvement in a subset of patients, although its risk–benefit profile remains debated (Gao et al., 2018; Zhou et al., 2020). Similarly, pan-PPAR agonists such as lanifibranor have shown anti-fibrotic signals in Phase IIb/III trials for MASH. These agents should be classified as maturity level I (clinically translatable) for metabolic liver fibrosis, though their application specifically to periodontitis-associated fibrosis—and their potential interaction with oral inflammation—has not been investigated. Whether periodontal disease modifies the therapeutic response to these agents remains an open question requiring dedicated subgroup analysis or mechanistic nesting studies.

By contrast, LOXL2 inhibitors (e.g., simtuzumab) have failed to meet primary endpoints in Phase II trials for bridging fibrosis or cirrhosis, while targeting LSEC capillarization through VEGF modulation or NO donors remains at the preclinical stage, supported largely by mechanistic studies in animal models (Wang and Liu, 2021; Wang et al., 2021). The bile acid–FXR/TGR5 axis, though mechanistically attractive, presents context-dependent effects: TGR5 agonism may be anti-fibrotic in MASLD/MASH but pro-fibrotic in cholestatic conditions, complicating therapeutic translation (Keitel and Häussinger, 2018; Ma et al., 2021; Spatz et al., 2021; Tang et al., 2026; Xiang et al., 2026). These strategies should be classified as maturity levels II–III, requiring further preclinical optimization and careful patient stratification before clinical testing. In summary, the three-phase framework identifies multiple theoretically actionable nodes, but their translational readiness varies widely. Clinically actionable strategies (level I) principally include FXR and PPAR agonists, which are already available for metabolic liver fibrosis, but their efficacy specifically in periodontitis-associated disease requires dedicated investigation. Preclinically validated strategies (level II), including MyD88 inhibitors, TLR4 antagonists, and barrier protectants, are mechanistically compelling but require safety and efficacy demonstration in human trials before any clinical recommendation. Conceptual strategies (level III), such as anti-gingipain vaccines and LSEC-targeted therapies, remain distant from clinical application. We emphasize that none of these strategies can currently be recommended for standard clinical care of periodontal disease-associated liver fibrosis, except for routine periodontal management as part of overall dental hygiene. The stratification proposed here is intended to guide future research prioritization: direct resources first toward level I and II strategies with the greatest potential for near-term clinical translation, while level III strategies warrant continued mechanistic exploration but should not be positioned as imminent therapeutic options. This evidence-transparent approach aligns with the broader evidence-tiering principle we have applied throughout this review and provides a realistic framework for clinicians and researchers to assess the maturity of each intervention candidate.

## 7 Conclusion

Periodontal disease, long viewed as a localized oral inflammatory condition, has emerged as a modifiable systemic inflammatory driver that may accelerate liver fibrosis through the interconnected oral-gut-liver axis. The pathogenic mechanisms elucidated in this review suggest a tripartite cascade: periodontal pathogens disseminate and activate hepatic TLR/NF-κB signaling; the inflammatory burden is then amplified through intestinal barrier disruption and endotoxin translocation; and finally, liver-intrinsic mechanisms take over to maintain fibrosis independently of external triggers. This three-phase framework not only integrates current evidence but also provides a rational basis for designing phase-specific, multi-node intervention protocols. Extensive epidemiological evidence demonstrating a dose-response relationship between periodontal disease severity and liver fibrosis stage reinforces the clinical significance of these mechanistic insights. However, translating these findings into clinical practice faces three interconnected challenges: technical bottlenecks in precisely tracking pathogens across anatomical niches, persistent residual confounding in observational studies that limits causal inference, and the absence of high-quality randomized controlled trials with liver histological reversal as a primary endpoint. The highest priority for future research is to establish interdisciplinary oral-liver follow-up cohorts and conduct rigorously designed multicenter trials. Deepening this interdisciplinary perspective may eventually integrate routine dental assessment and periodontal intervention into the comprehensive care of patients with chronic liver disease, potentially slowing fibrotic progression and improving long-term prognosis if ongoing and future trials confirm their efficacy. The oral-gut-liver axis thus represents not merely a scientific frontier but a tangible opportunity for multidisciplinary patient care.
